# Ecological and health risk assessment of trace metals in water collected from Haripur gas blowout area of Bangladesh

**DOI:** 10.1038/s41598-021-94830-0

**Published:** 2021-08-02

**Authors:** M. Farhad Howladar, Md. Numan Hossain, Khaleda Akter Anju, Debjani Das

**Affiliations:** 1 Department of Petroleum and Mining Engineering, Shahjalal University of Science and Technology, Sylhet, Bangladesh; 2 Department of Geography and Environment, Shahjalal University of Science and Technology, Sylhet, Bangladesh

**Keywords:** Ecology, Environmental sciences, Hydrology

## Abstract

The study aims to assess the trace metals and physicochemical properties of water in the adjacent to the Sylhet gas blowout area. Trace metals were analyzed using atomic absorption spectrophotometer, whereas physicochemical parameters were evaluated in-situ state using portable instruments and also in the laboratory. Trace metals Pb, Cd, and Ni were found in the water samples higher than the acceptable limit by WHO standards, whereas the concentration of Cu and Zn were within acceptable limit, respectively. The correlation coefficient matrix and factor loading analysis spectacle that the interrelationship among the physicochemical parameters, trace elements, as well as other ions are moderate to strongly corellated which reflecting the homogeneous source of origin. According to contamination factor, Nemerow multi-factor index, pollution load index, and also, potential ecological risk index, the water of the region is quite polluted in case of Pb, Cd, and Ni but unpolluted for Cu and Zn. The water quality index indicates that treatment of water is required before using it for domestic purposes. The health quotient and hazard index results are less than standard value 1 suggesting that there is no noncarcinogenic risk in the area. The carcinogenic analysis shows that the lifetime incremental cancer risk mean value of Cd and Ni are fairly insignificant and Pb is more significant for children to cause health problem. The ILCR value of Cd and Ni are insignificant whereas Pb is significant to pose health risk for adults. Physicochemical parameters revealed that the water was slightly acidic and soft in nature implying to avoid the water from this area for drinking purposes. At the end, it can be concluded that this study will be useful for the residence as well as the policymaker to take the protective surveillance measures around the areas.

## Introduction

Water is an important component of earth which is much essential for our daily life. Based on use, water can be typified as drinking, domestic, livestock, irrigation, industrial and so on. Currently, the quality of water is degrading visibly in different areas in Bangladesh as well as in the world. The anthropogenic activities such as rapid industrialization, irrigation, shipping, extracting mineral resources from the earth’s ground and others are responsible for it. Among them industrial operations associated pollution are mostly palpable. However, it is well known that the blowout of gas is one of the severe accidents in petroleum industry causing the degradation of various environmental components such as soil, water, air, etc. Moreover, the uncontrolled underground releases of natural gas are a vital issue to increase methane concentration in groundwater, surface water, soil, and air in the atmosphere^1^.

**Figure 1:**
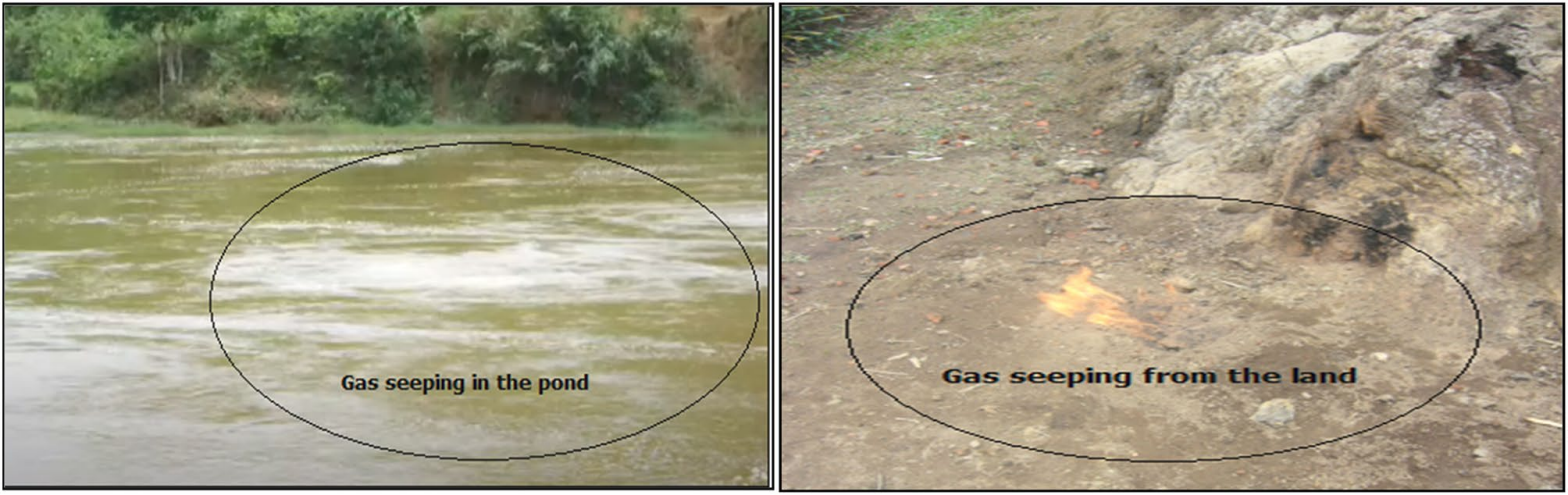
The seeping of natural gas by groundwater, surface water and the land in the area.

Haripur gas field was discovered in Sylhet region by the Pakistan Petroleum Limited (PPL)^2^. The country’s first drilling activities took place at Haripur (1955) in Sylhet where 6 wells were drilled by PPL. Among them, only two (Syl-3 and Syl-6) wells became operative^2,3^. During these drilling operations, the first detection of gas in the country took place in Haripur^4^. While conducting the operation, the blowout occurred in Syl-1 and Syl-4 well because of abnormal high pressure^5^. As a result, the gas has been blowing out forcefully, so damaged the total structures and environment of that area. Still, endlessly gas is seeping from this area from that time (Fig. 1).Thus, this study deemed that seeped methane changes the concentration level of trace elements with physicochemical properties of ground-surface water around the adjacent areas. Ultimately, this might have an impact on the public health and ecological system in the area. In addition, the natural gas leaks in freshwater aquifers can affect groundwater quality by modifying redox conditions, increasing in pH; declining in Eh; decreasing iron and manganese oxides and forming alkalinity by methane oxidation and many more^1^.

About 1.2 billion people are suffering from contaminated drinking and domestic use of water in the world^6^. Higher levels of pollutants in water are largely responsible for increasing threat toecology, human health, and the environment^7^. The health and the ecological system may have a considerable risk by transferring TMs in water to different plants to social health^8,9^.At present, trace elements effluence in water is the most concerned issue for the scientist as it crafts the serious health and ecological problems in nature^10,11^.Toxic metal-associated water pollution, health, ecological and environmental risk also mentioned by various authors^12–16^. The moderate to high ecological risk and heavy metal pollution was found in a lithofacies of subsurface well as well in the drilling crew and people in a hydrocarbon drilling environment^17^.Trace metals are the most common aquatic contaminants and responsible for creating a toxic environment^18^. TMs pollutions have turn into an attractive issue in the world because of its high diligence, toxic and carcinogenic characters^19^. The carcinogenic risks of heavy metal were found to have higher than the standard limit in several studies in Bangladesh which might have increase the cancer risk exposure to different ages of people^20^.Particularly, Nadim et al.^21^ and Wang et. al.^22^ entailed that environmental water pollution due to the operation of oil gas industries is very frequent in the developing country as well as in the Globe.

The Sylhet region has some previous studies on geology, structure, and blowout impact on environments are performed by Hiller and Elahi^23^, Khan et al.^24^, Faruque^25^, Khan and Nasir^26^. A number researchers like Howladar and Rahman^27^, Mamun et al.^28^, Numanbakth et al.^29^, Rahman et al.^30^ and others are carried out the research works to evaluate the impact of mining sector, gas field and rock crushing sides on ground-surface water chemistry including trace metals with another properties. However, no studies have been found on the impact of the blowout on the aquatic environment or the toxicity of TMs in surface and ground waters in the region. Thus, this study assessed (1) some physicochemical parameters and the trace metals (Ni, Cd, Cu, Zn, and Pb) that would have accumulated in the water body of the adjacent areas due to the blowout, (2) the ecological and human health risk coupled with TMs and the physicochemical properties of water, (3) the long-term impact of the massive gas blowout on humans and ecology, (4) the possible pollution intensity for effective management of water resources around the gas blowout areas of the Sylhet gas field. Finally, the present article on ecological-health risk evaluation will be encouraging to the proper authority and policy maker for understanding the highly risky areas to look after the overall ecosystem and human health professionally.

## Materials and methods

### Outline of the study region

The study was conducted in and around the blow out zones of the Haripur gas fields of Jaintapur Upazila which lies between the geographical coordinates 24.58′36″N′N to 24.58′57′N (latitudes) and 92.01′35″E′E to 92.01′57′E (longitudes).The Upazila is divided into six administrative units and 70 villages. The total population of this area is about 1,21,458^31^. This area is bounded by Meghalaya in the north, Sylhet city, Kanaighat-Golapganj and Kanaighat in the west, south and east, respectively. The prominent Jaflong Hills range is situated closely to the study area. The area lies within Sylhet Basin agro ecological zone. The weather is mostly humid and heavily dependent on the northern hilly region with a tropical monsoon climate. Haripur gas field is operated by Sylhet Gas Fields Limited, is a pioneer gas and oil Production Company in the country. The blowout accident took place on well Syl-1 and 4 in 1955 and 1962, respectively^26^. Accordingly, the drilling rig was destroyed thoroughly, forming a large deep hole. Successively, the hole was filled with water and made a large pond in the area. Finally, this well was abandoned, but still, gas is seeping continuously from fissures and hillsides surrounding the wells are shown in Fig. 1.

### Water samples and analysis

**Figure 2:**
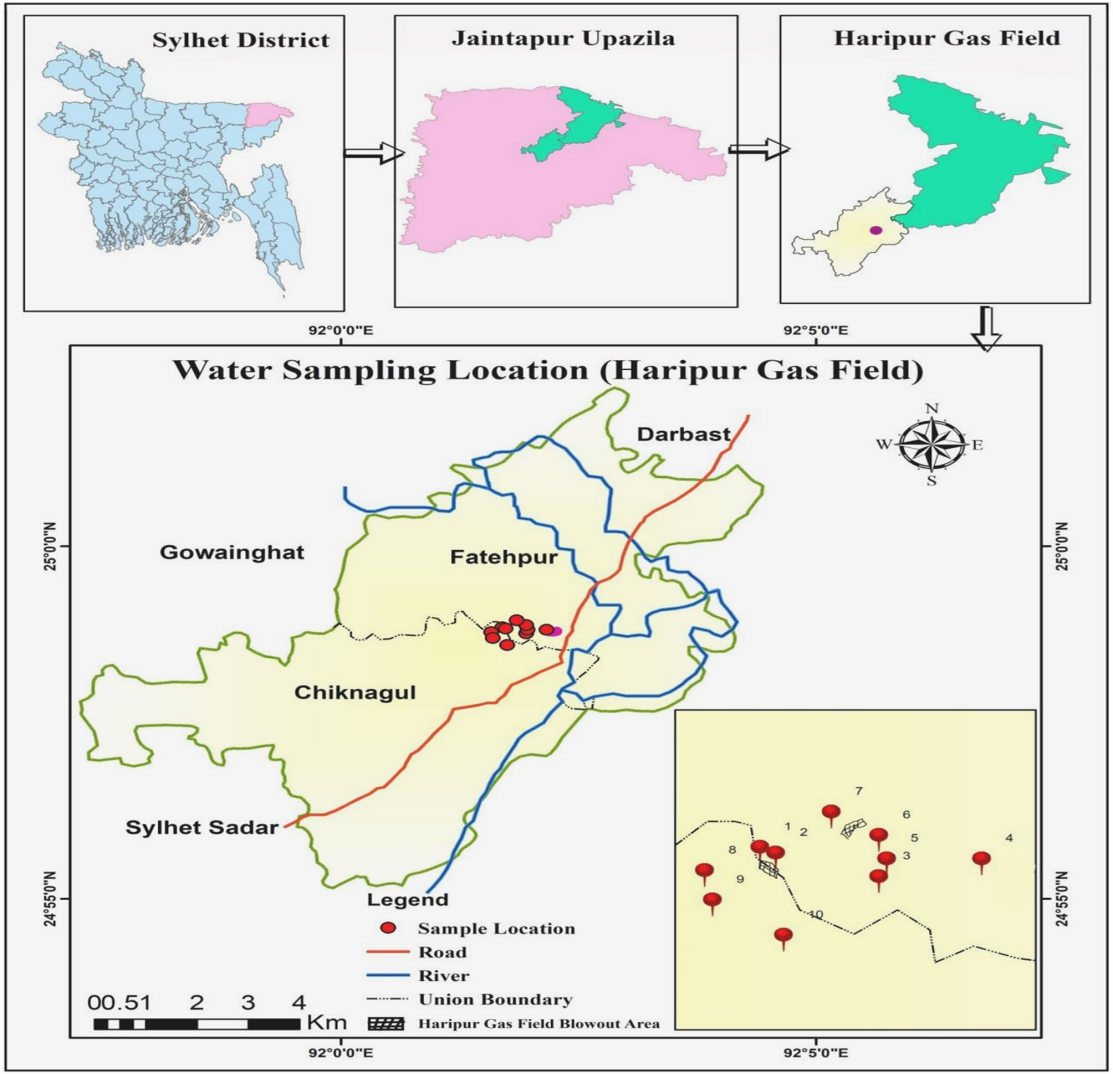
Water sampling locations of the study area.

Water samples were collected from10 different locations around the blowout area is shown in Fig. 2.Then, the samples took in plastic bottles and wrapped adequately with adhesive tape. The dry, clean and sterilized 2 L plastic bottles were used to carry these samples. After that, samples were brought to the laboratory for analysis. Proper methods and prerequisites have been fulfilled for analysis. Meanwhile, subjective statistical analyses were performed utilizing the given raw data to obtain the results. Besides, physicochemical parameters were undergone in-situ measurements. In this case, the pH and Oxidation Reduction Potential (ORP) were tested under HANNA potable Instruments HI 9126. The Electrical conductivity was measured by HANNA EC 215, whereas the turbidity with microprocessor turbidity meter HI 93703. The concentrations of carbon dioxide, chloride and Total alkalinity were measured by using titration method. The parameters such as total dissolved solids (TDS), total hardness (TH), and Ca^2+^ have been determined using EDTA titration method. These tests were performed in the laboratory of the Department of Petroleum and Mining Engineering (PME), Shahjalal University of Science and Technology (SUST), Sylhet, Bangladesh. In addition, the water samples have been digested with concentrated HNO_3_ for trace elements analysis in the laboratory of PME in SUST. After that, the samples were tested in the Soil Research Development Institute (SRDI) in Sylhet to determine Ni, Cd, Cu, Zn and Pb concentrations in water of the area. The concentrations of trace elements, including Ni, Cd, Cu, Zn, and also Pb were estimated with atomic absorption spectrophotometer (Model: AA-7000, Shimadzu Corporation, Japan) in SRDI, Sylhet. Completing the laboratory analysis, the descriptive statistical analyses were performed by MS Excel (2013). The interrelationship among different water quality parameters were attained by Correlation Matrix^27,32^ and factor analysis^32,33^. In this study, correlation and factor analysis were performed by using IBMSPSS 20. Moreover, the study area map has been constructed using ArcGIS Software, Version 10.3 of 2014. Primarily, the base map was collected from Local Government Engineering Department (LGED) of Bangladesh where they provide open access permission to use it^34^. To bring out the impact of blowout over the areas, the study employed several indices and spatial distribution map to visualization current situation around the area. The risk assessment can profoundly demonstrate the effect of an event on humans and other systems. This study evaluated the ecological risk as well as the health risk from the presence of trace elements (TMs) in the water around the gas field blowout zone. Subsequently, spatial distribution made it more precise through the lens of space.

### Water quality index

WQI is a useful way to achieve the nature of water in any region. In this study, WQI was calculated using parameters such as pH, EC, TDS, TH, turbidity, Pb, Ni, Cu, Cd. The computation of WQI has been conducted by the weighted arithmetic index method^35^. For this computation, the following equations were used:

1 $${\text{WQI}} = \frac{{\sum Q_{n} W_{n} }}{{\sum W_{n} }}$$

2 $${\text{Q}}_{{\text{n}}} = \left[ {\frac{Vn}{{Vs}}} \right] \times 100$$

3 $${\text{Q}}_{{{\text{PH}}}} = \left[ {{\text{Vn}} - {\text{Vi}}/{\text{Vs}} - {\text{Vi}}} \right] \times 100$$

4 $${\text{K}} = \frac{1}{\sum Vs}$$

5 $${\text{W}}_{{\text{n}}} = {\text{k}}/{\text{Vs}}$$

where the parameters: Q = quality rating of nth water quality parameter; W = unit weight of nth water quality parameter; K = constant of proportionality; V_n_ = actual amount of nth parameter present; Vi = ideal value of the parameter [Vi = 0, except for pH (Vi = 7)]; Vs = standard permissible value for the nth water quality parameter.

The calculated WQI values are classified within a predetermined range where under 50 refers excellent water quality of grade 1, WQI range 51 -100 expresses good water quality of grade 2, WQI range 101–200 denotes poor water quality of grade 3, WQI range 201–300 states very poor water quality of grade 4, and last one, WQI range above 300 refers very bad water quality of grade 5. In that case, proper treatment would be necessary to use the water for the domestic purpose^34^.

Moreover, this study considered the contamination factor^36–38^; pollution load index (PLI)^36,39^; Nemerow multi-factor index (PI)^36,40,41^. The ecological risk index (RI)^42,43^ and Heath risk assessment (including Carcinogenic risk as well as Noncarcinogenic risk) were determined as follows. The obtained result from the indices would make significant and specific adjusted output to the system by considering several indicators.

### Assessment of health risks using noncarcinogenic and carcinogenic risk analysis

The health risk depends largely on trace elements in water. Its assessment may be categorized as Carcinogenic or Non-carcinogenic^44^. The hazard quotients (HQ) as well as hazard index (HI), and also Incremental lifetime cancer risk (ILCR) were calculated to evaluate carcinogenic or noncarcinogenic risk from trace elements of water sample in the investigated region. It is known that carcinogenic or noncarcinogenic pollutants are exposure hazards sustained in nature for a longer time. From this perspective, the Chronic Daily Intake (CDI) has been determined using equations developed by the Environmental Potential Agency (US EPA) as follows:

6 $${\text{CDI}}_{{{\text{ingestion}}}} = \frac{{{\text{Cw}}*{\text{DI}}*{\text{ABS}}*{\text{EF}}*{\text{EP}}}}{{{\text{BW}}*{\text{AT}}}}$$

7 $${\text{CDI}}_{{{\text{dermal}}}} = \frac{{{\text{Cw}}*{\text{SA}}*{\text{Kp}}*{\text{ABS}}*{\text{ET}}*{\text{EF}}*{\text{EP}}*{\text{CF}}}}{{{\text{BW}}*{\text{AT}}}}$$

where C_w_ = concentration of toxic metals(mg/L), K_p_ = coefficient of permeability (cm/hour), ABS = factor of dermal absorption (unitless), DI = the amount of daily average intakewater (L/day), ET = time of exposure(h/event), ED = exposure period (years), EF = exposure frequency per annual (days/year), CF = conversion factor (L/cm3), BW = individual weight (Kg/individual), SA = skin area available for contact (cm^2^), and AT = average time (days).

Table 1 represents the estimated value of inputs for assessing chronic daily intake via oral and dermal ingestion^44,45^. A response dose and cancer slope factor values are presented in Table 2.

**Table 1:** The assumed value for CDI evaluation.

Parameters	Units	Value	
		Child	Adult
Concentration of trace element	µg/L	–	–
Daily average intake	L/day	1.8	2.2
Skin surface area	cm^2^	6600	18,000
Exposure time	h/event	1	0.58
Exposure period	years	6	70(Ingestion) 30(Dermal)
Exposure frequency	Day/year	365(Ingestion) 350(Dermal)	365(Ingestion) 350(Dermal)
Average time	days	2190	25,550
Conversion factor	L/cm^3^	0.001	0.001
Individual weight	kg	15	70
ABS	All	0.001	0.001
Coefficient of permeability	cm/h	Pb, Cu, Cd, Zn = 0.006, Ni = 0.0002	Pb, Cu, Cd, Zn = 0.006, Ni = 0.0002

**Table 2:** The response dose and cancer slope factor value^44,45^.

Elements	RfD oral	RfD dermal	CSF
Pb	1.4	0.42	8.5
Ni	20	5.4	0.84
Cd	0.5	0.005	6.1
Cu	40	12	–
Zn	300	60	–

The Health Quotients (HQ) evaluated by the following equation where CDI and RfD represented chronic daily intake and reference dose (both in mg/kg day), respectively.

8 $${\text{Health quotients: HQ}} = \frac{CDI}{{RfD}}$$

and the sum of the HQ represents the total potential health risks (HI)

9 $${\text{HI}} = \sum {\text{HQ}}$$

In the case of the carcinogenic risk evaluated with the formula mention by Mohammadi et al.^44^.

10 $${\text{ILCR}} = {\text{CDI}}*{\text{CSF}}$$

Here factor, CSF represents as Cancer slope factor (measured in mg/kg day) of trace elements in the water. In principle, the computed ratio of HI is greater than 1 indicates the possible toxic hazards, while the ratio less than 1 denotes no visible toxic effect on human health in the study area.

## Outcomes and discussion

### Physiochemical characteristics of water in the blowout region

The physiochemical properties of water were measured in the laboratory. The analyzed properties are shown in Table 3.

**Table 3:** The analyzed physiochemical properties of water.

Samples	pH	CO_2_ (ppm)	Turbidity (FTU/NTU)	Conductivity (µS/cm)	TDS (ppm)	Alkalinity (ppm)	Total Hardness (ppm)	Ca^2+^ (ppm)	ORP (mV)
SS-1	6.63	2	39.8	33	21.12	115	75	30.06	40.1
SS-2	6.51	6	20.1	35.3	22.59	130	45	18.04	35.6
SS-3	6.54	7	10.8	150.1	96.06	170	35	14.03	34
SS-4	6.38	7	9.6	103.8	66.43	100	40	16.03	42.9
SS-5	6.69	9	4.9	105.9	67.78	90	30	12.02	26.8
SS-6	7.5	12	1.2	N/A	N/A	195.24	185	74.15	− 17.5
SS-7	6.4	5	0.6	59.1	37.82	70	20	8.02	43
SS-8	6.37	3	0.8	64.8	41.47	75	15	6.01	43.4
SS-9	6.45	10	0.5	112.8	72.19	60	30	12.02	39
SS-10	5.82	4	10.3	25.8	16.51	85.71	15	6.01	73
Max	7.5	12	39.8	150.1	96.06	195.24	185	74.15	73
Min	5.82	2	0.5	25.8	16.51	60	15	6.01	− 17.5
Ave	6.529	6.5	9.86	76.73	44.19	109.1	49	19.63	36.03
St. dev	0.42	3.17	12.24	43.21	27.66	44.34	50.92	20.41	22.35
WHO standard (2011)	7–8	–	< 5	0–800	< 500 ppm	120 ppm	300 mg/L	–	–

*N/A* not available.

The average value of pH is 6.529 indicates water of the study area is slightly acidic in nature. The average value of CO_2_ (6.5) complied with the lowering tendency of pH. The average ORP value 36 also reflecting the sign of acidic water in the study region. According to WHO standards (2011), the value of conductivity within range 0–800, Total dissolved solids less than 500 ppm, alkalinity 120 ppm, and total hardness less than 300 mg/L are allowable for drinking and domestic purpose^37^. The average value of conductivity 76.7 µs/cm, total dissolved solids 44.2 ppm, alkalinity 109.1 concurred with the dirking water standard by WHO (2011). The average value of TH is 49 ppm points out that the properties of water are soft.

### Spatial distribution of trace elements derived from water bodies around the blowout area

**Figure 3:**
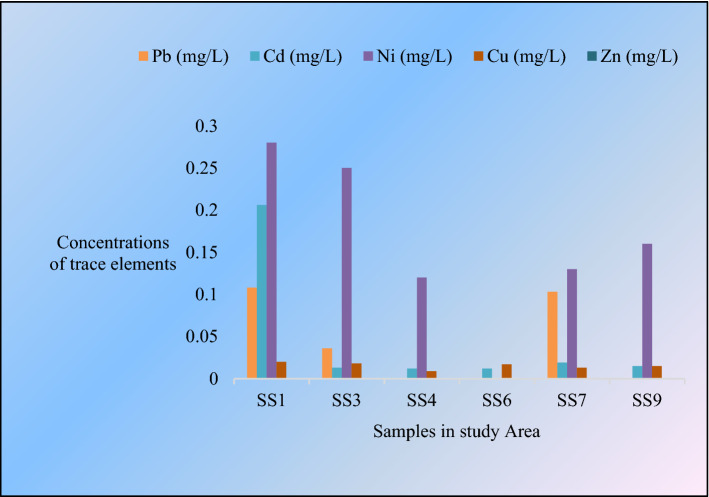
The concentration of trace elements in the study area.

**Figure 4:**
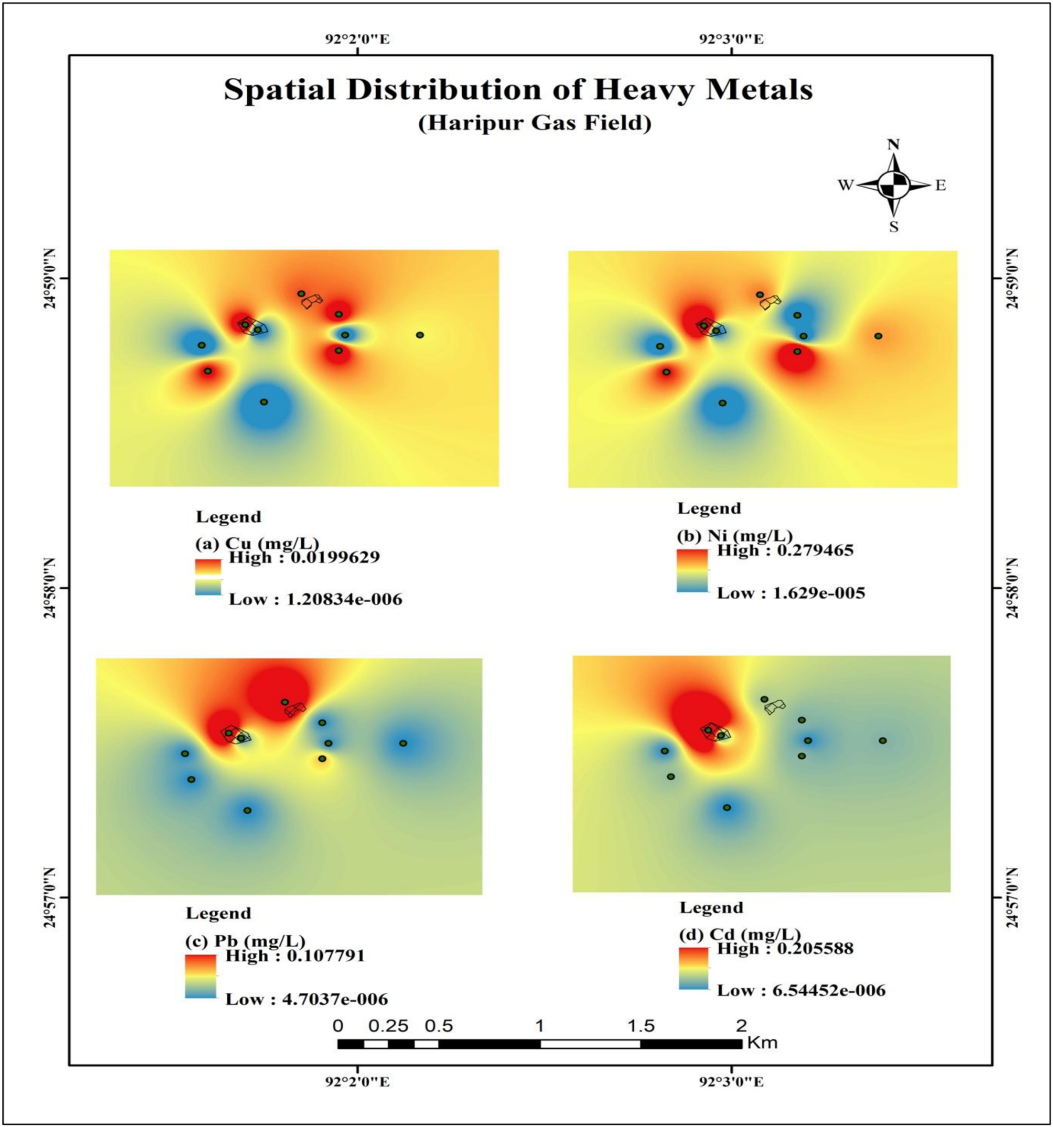
The spatial distribution map of toxic metals in the area.

The primary purpose of this study is to understand the concentration level of different Trace metals in the area. In this study, Pb, Ni, Cu, Cd, and Zn were examined (Fig. 3). Besides the toxic metal spatial distribution map is constructed using the inverse distance weighting (IWD) method in Arc GIS (version 10.5). The map (Fig. 4) shows common patterns of hotspots near the Syl-1 blowout area for every metal. This scenario indicates that these metals are originated from the same source^46^.The contiguous area near Syl-4 well also exhibited a similar pattern to Syl-1 for all metals except Cd. A high concentration of Cd was found closed to the Syl-1 area. The elevated concentration of toxic metals like Ni, Pb, and Cd are found in the adjacent areas of blowout points (Fig. 4). Continuous gas escaping from these abandoned wells might stimulate the trace metal accumulation, especially Pb would be more toxic when it will come to a contact with gasoline (Syl-1 and Syl-4)^7^. The non-essential toxic metals like Ni, Cd, and Pb in water can pose a serious health threat inthesite^47^. In addition, these toxic elements can contribute to acute or chronic health issues like high blood pressure, kidney failure, headache, abdominal pain, cancer, nerve damage, and so on for the long-term consumption of such water^48^.Standard value of Pb in water is 0.01 mg/L, Ni is 0.02 mg/L, Cu is 2 mg/L, Cd is 0.003 mg/L in water^37^. In this analysis, the average value of Pb = 0.04, Cd = 0.05, Ni = 0.16, Cu = 0.03 mg/L, respectively. The TMs like Zn concentration is about zero or below the detection level for water samples in the study location. The values of Pb, Cd and Ni were higher than the standards level indicates that the water should not be used for any purpose^49^.

### Correlation coefficient (R) matrix of water quality parameter presented in the blowout area

A Correlation matrix represents the relationship among several variables. It is generated based on the correlation coefficient, which ranges from − 1 to 1. The value of correlation coefficient (1, − 1) indicates perfect correlation, (− 0.9 to − 0.7 or 0.9–0.7) shows strong correlation, (0.4–0.6 either positive or negative) represents moderate correlation, (0.1–0.3 or − 0.1 to − 0.3) displays as weak and 0 indicates no relationship between variables^50^. The mathematical expressions are described in the article by MacMillan et al.^51^ to evaluate the correlation coefficient (r).

The correlation matrix is shown in Table 4. From the Table 4, it is clear that the pH shows a moderate to strong correlation with CO_2_ (0.63) and alkalinity (0.69). Whereas, it shows a very strong positive correlation with Total Hardness (TH) and Ca^2+^ (0.88), respectively. The moderately positive correlation reflects with EC (0.41) and trace elements Ni (0.62). The rest of parameters show a negative correlation. The CO_2_ exhibits a good correlation with EC (0.72), TH and Ca^2+^ (0.52). Alkalinity states a good correlation with TH and Calcium ions (0.76). EC shows a maximum correlation with TDS (1.00); maximum correlation also found in the case of TH and Ca^2+^. Turbidity has a positive correlation for all of the parameters except EC and TDS. TDS shows a strong positive correlation with all of the trace elements, in the case of Pb (0.54), Cd (0.88), Ni (0.68) and for Cu the value is 0.64. All trace elements have a strong correlation with each other. Pb represents a good correlation with Cd (0.65), Ni (0.54) and Cd (0.35). Ni has a strong correlation with Cd (0.61), Cu (0.43). Cu also implies a good correlation with Cd (0.58). In the end, it can be mentioned that a strong positive correlation can be detected among all of the trace elements and also for most of the relative parameters. CO_2_ established the equilibrium state in the water with ions might be lowering the oxidation. The trace metals Cu and Cd were positively correlated with the turbidity. The washed turbid water from the blow out areas might stimulate these trace metals. The inverse association with oxidation and total hardness indicates the less vegetated areas have higher influx rate of soil materials. It implies the result of the correlation matrix indicated that all of the trace elements and also relevant ions presented in the water of blowout area resultant from the same source^46^.

**Table 4:** Correlation coefficient matrix of water parameters.

	pH	CO_2_	Turbidity	EC	TDS	Alk	TH	Ca^2+^	ORP	Pb	Cd	Ni	Cu
pH	1												
CO_2_	0.63	1											
Turbidity	− 0.07	− 0.54	1										
EC	0.41	0.72	− 0.44	1									
TDS	− 0.22	0.18	− 0.24	1.00*	1								
Alk	0.69*	0.38	0.19	0.26	− 0.19	1							
TH	0.88**	0.52	0.07	− 0.13	− 0.52	0.76*	1						
Ca^2+^	0.88**	0.52	0.07	− 0.13	− 0.52	0.76*	1.0**	1					
ORP	− 0.98**	− 0.71*	0.19	− 0.54	0.18	− 0.68*	− 0.86**	− 0.86**	1				
Pb	− 0.17	− 0.48	0.54	− 0.50	− 0.13	− 0.62	− 0.27	− 0.27	0.29	1			
Cd	− 0.08	− 0.75	0.88**	− 0.58	− 0.33	− 0.23	0.06	0.06	0.28	0.65*	1		
Ni	0.16	− 0.48	0.68	− 0.75	− 0.60	0.13	0.19	0.19	− 0.04	0.54*	0.61*	1	
Cu	− 0.08	− 0.49	0.64*	− 0.91*	− 0.69	− 0.37	0.16	0.18	0.18	0.35*	0.59*	0.43*	1

*Correlation is significant at the 0.05 level.

**Correlation is significant at the 0.01 level.

### Factor loading of water parameters

The interrelationship within a set of variables or objects is represented by factor analysis. The factors contain all of the basic information about a wider set of variables or observed objects. It shows how the variables are strongly correlated with the determined factor. Factor analysis is also known as a multivariate approach to reducing data^33^. Among different types of factor analysis, Principal component analysis account for the maximum variance of observed variables. So, it can be called variance-oriented^33^. Factor loading shows how certain variables strongly correlate for a given factor. Factor loading varies from − 1 to + 1 where the value of factor loading below − 0.5 or above 0.5 suggested good correlations and value closed to − 1 or + 1, suggesting a more robust correlation^32^. The Table 5 represented the principal component analysis result of factor solution.

**Table 5:** Principal components analysis results of water parameters.

Water parameters	Components	
	1	2
PH	0.239	0.939
CO_2_	− 0.923	0.242
Turbidity	0.966	− 0.089
EC	− 0.876	− 0.033
TDS	− 0.876	− 0.033
Alkalinity	− 0.173	− 0.266
TH	0.965	− 0.143
Ca^2+^	0.965	− 0.143
ORP	0.436	− 0.875
Pb	0.572	0.773
Cd	0.895	0.105
Ni	0.765	0.378
Cu	0.829	− 0.227

Extraction method: principal component analysis and only 2 components extracted.

From analysis (Table 5), it can be realized that the water quality parameters such as Turbidity, TH, Ca^2+^, Cd, Ni and Cu have a stronger correlation with each other’s reflecting their source of origin might be from the same area^14^. Factor loading also suggested that more robust interconnection exists among CO_2_, EC and TDS. In this analysis, the two-factor solution explained approximately 80.6% of the variance. The eigenvalue, total variance explained are represented in Supplementary Table S1. That percentage is high enough to accept the results. It can also be added that the red and yellow colored loading represented strong correlation with each other^46,52^.

### Water quality index (WQI)

The WQI is one of the best tools for monitoring the surface-groundwater contamination and can be used for water quality improvement programs. The WQI is determined from various  physicochemical parameters like pH, EC, TDS, TH, EC, and so forth. Higher estimation of WQI indicates poor water quality and lower estimation of WQI shows better water quality. During this examination, WQI esteems a range from 0.02633 to 5144.37 and are characterized into five water types shown in Table 6. The noteworthy WQI is recorded in case of (sample-1) which demonstrates an elevated level of contamination. Water sample 2, 5, 8 and 10 are grouped under class-1 which demonstrates there is a lower degree of pollution in water. In addition, WQI calculation for sample 2, 5, 8 and 10 excluded trace elements value and WQI evaluation for sample 1, 3, 4, 6, 7 and 9 included the heavy metals value in water. These results also clarify the association of heavy metals on water quality degradation of the study area.

**Table 6:** Classification of the water quality index for individual parameter of water.

Samples	WQI	Remarks	Category for water
1	5144.3	Proper treatment required before use	5
2	0.17	Excellent	1
3	1223.7	Proper treatment required before use	5
4	655.55	Proper treatment required before use	5
5	0.06	Excellent	1
6	231.09	Very poor	4
7	1004.1	Proper treatment required before use	5
8	0.03	Excellent	1
9	854.71	Proper treatment required before use	5
10	0.09	Excellent	1
Avg	911.38	Proper treatment required before use	5

### The situation of contamination in the area

**Figure 5:**
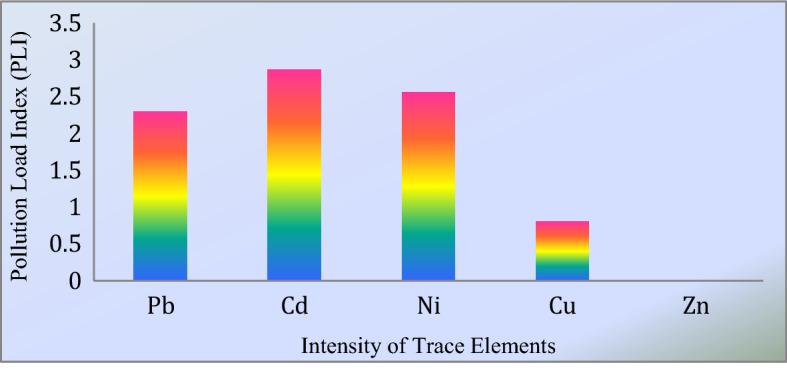
Pollution load index of the study area.

The level of contamination has been demonstrated in terms of the CFi, PLI, and also PI analysis of water samples around the blowout area. The values of CFi are indicated the degree of contamination. The intensity of CFi has been determined with some numerical values like 1, 3, and 6. The CFi value is less than 1, which implies low contamination, as the value is > 6 indicated a high degree of contamination^36^. The Table 7 elucidates that the degree of contamination in the case of trace elements Pb, Cd, and Ni are very high for most of the locations of the research sides. Besides Cu and Zn exhibit that level contamination is low in the area. In other cases, the PLI can be evaluated by using the CFi value. The value of PLI greater than 1 symbolizes polluted and less than 1 represents the unpolluted status^36,39^. The pollution load index rate of Pb, Cd, and Ni are 2.3, 2.87, and 2.56, respectively (Fig. 5).This result indicates the pollution of water bodies in the sampling sites. The other elements such as Cu and Zn are within the allowable limit are shown in Fig. 5. Moreover, the PI indicates similar results as CFi and PLI.

**Table 7:** Contamination factor of water samples.

Samples	Pb	Cd	Ni	Cu	Zn
**Contamination factor**					
SS1	10.80	68.67	14.00	0.01	–
SS3	3.6	4.33	12.50	0.009	–
SS4	–	4.00	6.00	0.0045	–
SS6	–	4.00	–	0.0085	–
SS7	10.30	6.33	6.50	0.0065	–
SS9	–	5.00	8.00	0.0075	–

### The state of potential ecological threat in the area

**Figure 6:**
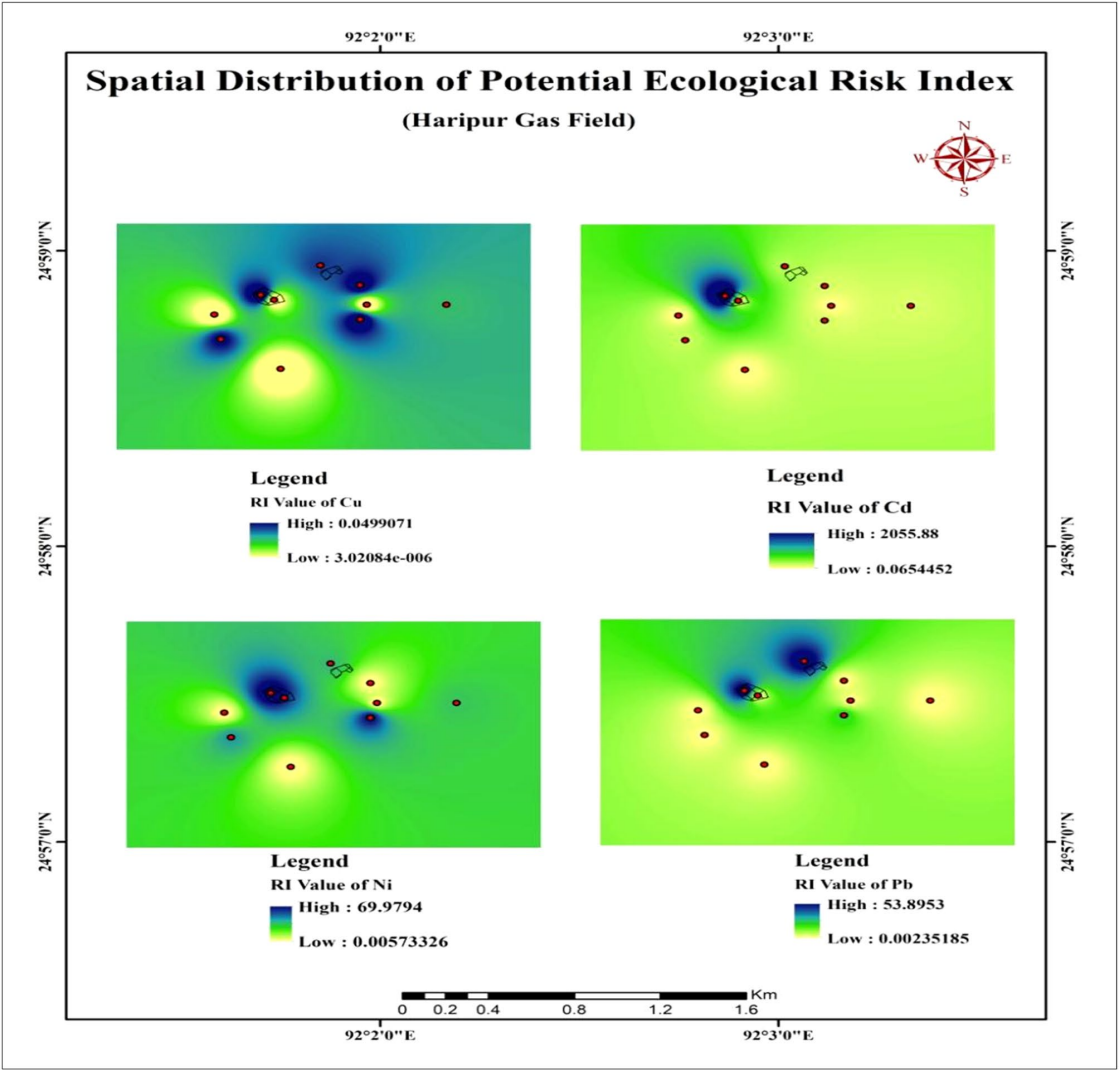
A map of the spatial distribution of potential ecological risk threats in the study area.

The ecological potential risk index has been appealed to detect the possible threat to the ecological system in the adjoining area. The calculated RI value provided the risk factor of water for understanding the ecological threat. When the RI value is more than 600, it is considered a polluted case^11,14,36^. The computed RI value of the study for Pb, Cd, Ni, and Cu are 123.5, 2770, 235, and 0.23, respectively (Table 8). The value of Cd is high enough (RI > 600). So, the Cd values indicated that the potential threat to the ecological system. Besides, the TMs like Ni and Cu are specified medium to low ecological pollution in the area are shown in Table 8. Moreover, the spatial distribution has been presented to outlook the potential ecological threats around the blow out location of the gas field is shown in Fig. 6.

**Table 8:** Ecological risk index (RI) of the study area.

Sample	Pb	Cd	Ni	Cu
**The potential ecological risk index**				
SS1	54	2060	70	0.05
SS3	18	130	62.5	0.045
SS4	0	120	30	0.023
SS6	0	120	0	0.043
SS7	51.5	190	32.5	0.033
SS9	0	150	40	0.033
RI	123.5	2770	235	0.190

The spatial distribution map of RI also pointed out the high ecological risk closed to the blowout areas (Fig. 6). From these results, it can be implied that the use of  this water for domestic or drinking purposes, can be harmful for living beings. Moreover, it can be distressed the ecological system in the site. Hence, the use of the water from this site should be avoided by dwellers near the blowout areas of the gas field.

### Assessment of noncarcinogenic health risks

Noncarcinogenic risk is one of the vital categories of human health risk assessment. It is known that a polluted environment is highly liable for causing a health risk. Toxic metal presents in water also very harmful for public health, including child and adult both. The health risks may be extended through ingestion and skin absorption of water. To know the harmful impacts of trace elements of water on the human body, noncarcinogenic risk evaluation is more important. For that, the value of CDI for ingestion and dermal absorption was evaluated at the beginning to identify such risk index (Supplementary Table S2 and S3). Then the CDI has been divided with the RfD value. From where, the HQ can be acquired separately for ingestion and dermal absorption. The summation of HQ_ingestion_ and HQ_dermal_ expressed the HQ_total_. And the HQ_total_ entirety was used to achieve the HI are shown in Table 9.

**Table 9:** The HQ and hazard index (HI) value of noncarcinogenic analysis of the area.

HQ total	For child					For adult				
Samples	Pb	Cd	Ni	Cu	HI	Pb	Cd	Ni	Cu	HI
SS1	8.99E−03	6.48E−02	1.63E−03	5.82E−05	7.55E−02	2.44E−03	1.55E−02	4.41E−04	1.58E−05	1.84E−02
SS3	3.00E−03	4.09E−03	1.46E−03	5.24E−05	8.59E−03	8.13E−04	9.77E−04	3.93E−04	1.42E−05	2.20E−03
SS4	–	3.77E−03	7.00E−04	2.62E−05	4.50E−03	–	9.01E−04	1.61E−04	7.12E−06	1.07E−03
SS6	–	3.77E−03	–	4.95E−05	3.82E−03	–	9.01E−04	–	1.34E−05	9.15E−04
SS7	8.57E−03	5.98E−03	7.48E−04	3.79E−05	1.53E−02	2.33E−03	1.43E−03	2.05E−04	1.03E−05	3.97E−03
SS9	–	4.72E−03	9.33E−04	4.37E−05	5.69E−03	–	1.13E−03	2.52E−04	1.19E−05	1.39E−03
Min	3.00E−03	3.77E−03	7.00E−04	2.62E−05	3.82E−03	8.13E−04	9.01E−04	1.61E−04	7.12E−06	9.15E−04
Max	8.99E−03	6.48E−02	1.63E−03	5.82E−05	7.55E−02	2.44E−03	1.55E−02	4.41E−04	1.58E−05	1.84E−02
Mean	6.85E−03	1.45E−02	1.09E−03	4.46E−05	2.41E−02	1.86E−03	3.47E−03	2.90E−04	1.21E−05	4.65E−03

The results elucidate that case of adult, the mean value of CDI_Total_ for Pb is 1.29E-03, Cd is 1.45E-03, Ni is 4.93E-03 and Cu is 4.83E-04, respectively. For the child, the mean value of CDI_Total_ in the case of Pb, Cd, Ni, and CU is4.7544E-03, 5.33E-03, 1.81E-03 and 1.77E-04, correspondingly. Additionally, the order of CDI_Total_ for adults are Ni > Cd > Pb > Cu (Supplementary Table S2) whereas, for child, it is quite different. In the case of children, the order  are Cd > Pb > Ni > Cu are presented in Supplementary Table S3.

The mean values of HQ_Total_ of Pb, Cd, Cu, and Ni are ranging from 4.46E−05 to 1.45E−02 for child. Besides, these values for adults are extending from 1.21E−05 to 4.66E−03. These values suggest that the trace elements in the water of the study area are quite harmful to the child than an adult. The children's HQ_Total_ has been ordered as Cd > Pb > Ni > Cu and for the adult Cu < Ni < Pb < Cd using the mean values of water in the blowout area. The minimum, maximum and mean values of HI for children are 3.82E−03, 7.55E−02, and 2.41E−02, respectively. And for adults, these values are 9.15E−04, 1.84E−02, and 4.65E−03. At this point, the mean value of HQs and HI of the study are less than 1, which defines that there is no adverse health-hazardous threat due to the exposure of toxic metals^53^. Hence from this computation, it can be concluded that noncarcinogenic risks are minor for the public health around the blowout area of Haripur Gas Field.

### Carcinogenic health risk evaluation

The excess TMs in water, soil or foods are blamed for causing the risk for ecology and health. These elements can cause cancer in the case of drinking such water for a long time. In this article, the values of ILCR of TMs in the areas are shown in Table 10. It is recognized that the ILCR value less than 1 × 10^–6^ can be considered as insignificant event to cause disease^11,44^. At this time, the risk of cancer can be neglected. Whereas, the values of ILCR more than 1 × 10^–4^ can be considered as significant to cause cancer like disease^11,14,44^.

**Table 10:** ILCR value of trace element presents in the water sample.

Samples	For adult			For children		
	Pb	Cd	Ni	Pb	Cd	Ni
**ILCR**						
SS1	2.89E−02	3.96E−02	7.39E−03	1.06E−01	1.45E−01	2.72E−02
SS3	9.64E−03	2.50E−03	6.60E−03	3.53E−02	9.16E−03	2.43E−02
SS4	–	2.31E−03	2.69E−03	–	8.45E−03	1.16E−02
SS6	–	2.31E−03	–	–	8.45E−03	–
SS7	2.76E−02	3.65E−03	3.43E−03	1.01E−01	1.34E−02	1.26E−02
SS9	–	2.88E−03	4.23E−03	–	1.06E−02	1.55E−02

**Figure 7:**
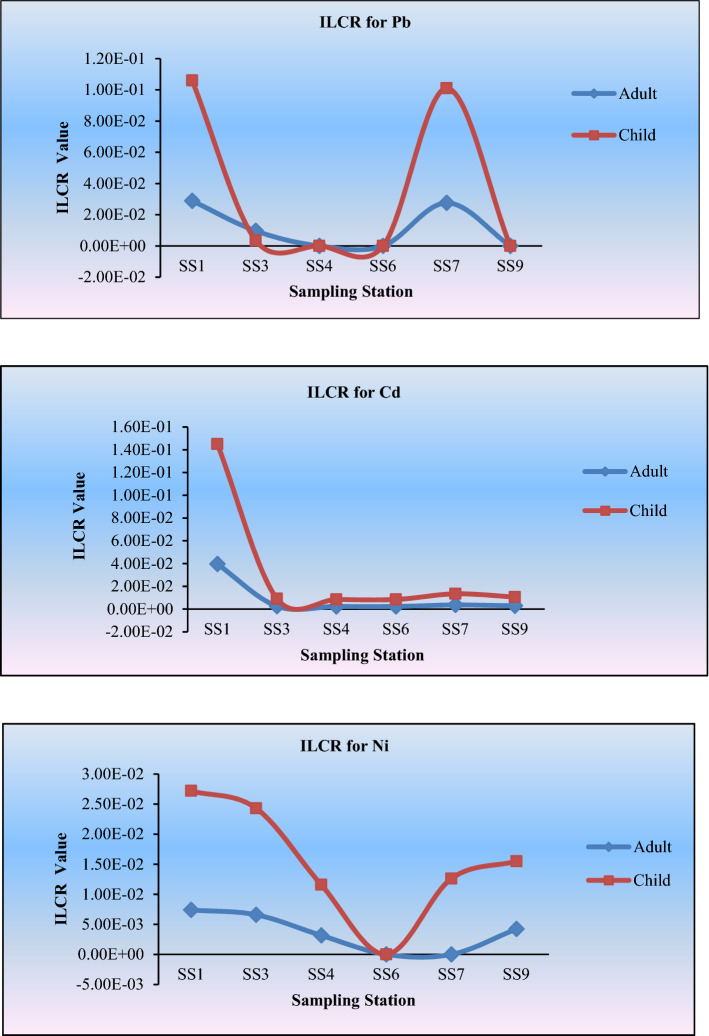
The incremental lifetime cancer risk (ILCR) for TMs in the blowout area.

The ILCR values of the trace elements are presented in Table 10.This Table also displays the mean, maximum, and minimum ILCR values for children as well as adults. The mean values of Pb, Cd and Ni for adults are 2.20 × 10^–2^, 8.87 × 10^–3^, 4.06 × 10^–3^, respectively. But, these values for children are 8.08 × 10^–2^, 3.25 × 10^–2^, and 1.82 × 10^–2^, respectively. The mean ILCR values of Cd and Ni (Fig. 7) are harmful and Pb values represented more harmful for children^45^. For Adults, the ILCR values of Cd and Ni are also significant and Pb is more noticeable to cause health risk (Fig. 7)^7,13,36,42^. The carcinogenic risk of these elements can be ranked as Pb > Cd > Ni.

A study carried out by Xu et al.^54^ where shown that the pollution status from an oil-based drill cuttings field. The result of that study revealed that the metal pollution was moderate and also noticed that the noncarcinogenic and carcinogenic risks of drilling workers were within the permissible level. The present research implied the same as that the blown-out areas were out of the health exposure except for children. Besides, another study led by Guan et al.^55^ in a mining area found an uneven distribution of potential ecological risk. However, this article addressed this gap considering risk assessment indices and spatial distribution of trace metals in a comprehensive way which could be a good foundation for future study in this field.

## Concluding remarks

The study assessed the ecological and health risks closed to the Haripur gas field well blowout area. The physicochemical properties and TMs such as Pb, Ni,Cd, Cu Zn were analyzed in the laboratory. According to physicochemical analysis, the water is slightly acidic and not suitable the drinking purpose. The computation of WQI indicated that proper treatment is necessary before using the water of the study area. The result of TMs analysis are shown that the average concentrations of Pb, Ni and Cd were within the WHO standard limit. Besides, Cu and Zn concentrations also belong to the permissible limit. The contamination factor values of Pb, Cd, and Ni are high, whereas Cu and Zn are low.

The correlation and factor loading show that the significant relationships among various parameters. The correlation of TDS with Pb (0.54), Ni (0.68), Cd (0.88), and Cu (0.64) reflected high interrelationship to one another. Also all TMs have a stronger correlation with each other. Furthermore factor analysis suggested stronger interconnection exists among turbidity, TH, Ca^2+^, Cd, Ni, Cu, and so on implied that all of the TMs may be originated from same source.

The pollution load index value for Pb (2.30), Cd (2.87), and Ni (2.56) show the pollution, whereas Cu (0.68) and Zn characterized unpolluted water bodies in the area. The multi-factor index also represents a quite similar indication with PLI. The potential ecological risk index for Cd (2770) is greater than 600, expressing the ecological pollution. But in the case of Ni (235), Pb (123.5) and Cu (0.23) implicate the negligible ecological pollution closed to blowout areas. The ILCR mean value of Cd, Ni, and Pb for an adult is higher than 1E-06 which stands fora significant risk. The value of Pb for an adult is more significant than others. On the other hand, the CDI and HQ value of TMs from the noncarcinogenic index are positioned as Cd > Pb > Ni > Cu > Zn. Besides, the values of the Hazard Index for the child is 1.89E−02 and for an adult is 4.65E−03.The mean value of HQ and HI are less than one implied that the noncarcinogenic health risk is negligible by exposing the TMs to public health through ingestion and dermal absorption. Nonetheless, this study discouraged the dwellers to drink this water from blowout areas. Additionally, the outcomes of the article will play a vial role to have the proper water pollution management, monitoring, and community awareness for controlling the ecological-health risk in and around the blowout as well as other industrial areas in Bangladesh.

## Supplementary Information

Supplementary Information.
