# Supplementary material for: Ecological and health risk assessment of trace metals in water collected from Haripur gas blowout area of Bangladesh
Source: Sci Rep. 2021 Aug 2;11:15573. doi: 10.1038/s41598-021-94830-0 (PMC8329277; doi:10.1038/s41598-021-94830-0)
Supplement: Supplementary file 1 — Supplementary Information. [file 41598_2021_94830_MOESM1_ESM.docx]

**Supplementary Tables**

Supplementary Table S1: Total variance explained by factor analysis.

| **Total Variance Explained** | | | | | | | |
| --- | --- | --- | --- | --- | --- | --- | --- |
| Component | Initial Eigenvalues | | | Extraction Sums of Squared Loadings | | | Rotation Sums of Squared Loadings^a^ |
|  | Total | % of Variance | Cumulative % | Total | % of Variance | Cumulative % | Total |
| 1 | 7.856 | 60.432 | 60.432 | 7.856 | 60.432 | 60.432 | 7.787 |
| 2 | 2.632 | 20.244 | 80.676 | 2.632 | 20.244 | 80.676 | 3.042 |
| 3 | 1.640 | 12.618 | 93.295 |  |  |  |  |
| 4 | .872 | 6.705 | 100.000 |  |  |  |  |
| 5 | 3.080E-016 | 2.369E-015 | 100.000 |  |  |  |  |
| 6 | 2.579E-016 | 1.984E-015 | 100.000 |  |  |  |  |
| 7 | 2.255E-016 | 1.735E-015 | 100.000 |  |  |  |  |
| 8 | 1.248E-017 | 9.596E-017 | 100.000 |  |  |  |  |
| 9 | -6.619E-017 | -5.091E-016 | 100.000 |  |  |  |  |
| 10 | -2.030E-016 | -1.562E-015 | 100.000 |  |  |  |  |
| 11 | -2.907E-016 | -2.236E-015 | 100.000 |  |  |  |  |
| 12 | -3.541E-016 | -2.724E-015 | 100.000 |  |  |  |  |
| 13 | -6.309E-016 | -4.853E-015 | 100.000 |  |  |  |  |
| Extraction Method: Principal Component Analysis. | | | | | | | |
| a. When components are correlated, sums of squared loadings cannot be added to obtain a total variance. | | | | | | | |

Supplementary Table S2. CDI Ingestion, Dermal absorption water quality parameter Total CDI for Adult.

| For Adult | CDI Ingestion(mg/kg-day) | | | | CDI dermal (mg/kg-day) | | | | CDI total (mg/kg-day) | | | |
| --- | --- | --- | --- | --- | --- | --- | --- | --- | --- | --- | --- | --- |
| Sample | Pb | Cd | Ni | Cu | Pb | Cd | Ni | Cu | Pb | Cd | Ni | Cu |
| SS1 | 3.39E-03 | 6.47E-03 | 8.8E-03 | 6.29E-04 | 6.62E-06 | 1.26E-05 | 3.43E-06 | 1.23E-06 | 3.4009E-03 | 6.49E-03 | 8.8E-03 | 6.3E-04 |
| SS3 | 1.13E-03 | 4.09E-04 | 7.86E-03 | 5.66E-04 | 2.21E-06 | 7.97E-07 | 3.06E-06 | 1.1E-06 | 1.1336E-03 | 4.09E-04 | 7.86E-03 | 5.67E-04 |
| SS4 | - | 3.77E-04 | 3.77E-03 | 2.83E-04 | - | 7.35E-07 | 1.47E-06 | 5.52E-07 | - | 3.78E-04 | 3.77E-03 | 2.83E-04 |
| SS6 | - | 3.77E-04 | - | 5.34E-04 | - | 7.35E-07 | - | 1.04E-06 | - | 3.78E-04 | - | 5.35E-04 |
| SS7 | 3.24E-03 | 5.97E-04 | 4.09E-03 | 4.09E-04 | 6.31E-06 | 1.16E-06 | 1.59E-06 | 7.97E-07 | 3.24E-03 | 5.98E-04 | 4.09E-03 | 4.09E-04 |
| SS9 | - | 4.71E-04 | 5.03E-03 | 4.71E-04 | - | 9.19E-07 | 1.96E-06 | 9.19E-07 | - | 4.72E-04 | 5.03E-03 | 4.72E-04 |
| Mean | 1.29E-03 | 1.45E-03 | 4.92E-03 | 4.82E-04 | 2.52E-06 | 2.83E-06 | 1.92E-06 | 9.4E-07 | 1.29E-03 | 1.45E-04 | 4.93E-03 | 4.83E-04 |

Supplementary Table S3. CDI Ingestion, Dermal absorption water quality parameter Total CDI for Children.

| For Children | CDI Ingestion(mg/kg-day) | | | | CDI dermal(mg/kg-day) | | | | CDI total(mg/kg-day) | | | | |
| --- | --- | --- | --- | --- | --- | --- | --- | --- | --- | --- | --- | --- | --- |
| Sample | Pb | Cd | Ni | Cu | Pb | Cd | Ni | Cu | Pb | Cd | Ni | Cu |  |
| SS1 | 1.24E-02 | 2.37E-02 | 3.22E-02 | 2.30E-03 | 4.56E-05 | 8.69E-05 | 1.18E-04 | 8.44E-06 | 1.2473E-02 | 2.38E-02 | 3.23E-02 | 2.31E-03 |  |
| SS3 | 4.14E-03 | 1.5E-03 | 2.88E-02 | 2.07E-03 | 1.52E-05 | 5.48E-06 | 1.05E-04 | 7.59E-06 | 4.1577E-03 | 1.50E-03 | 2.89E-02 | 2.08E-03 |  |
| SS4 | - | 1.38E-03 | 1.38E-02 | 1.04E-03 | - | 5.06E-06 | 5.06E-05 | 3.8E-06 | - | 1.39E-03 | 1.39E-02 | 1.04E-03 |  |
| SS6 | - | 1.38E-03 | - | 1.96E-03 | - | 5.06E-06 | - | 7.17E-06 | - | 1.39E-03 | - | 1.96E-03 |  |
| SS7 | 1.18E-02 | 2.19E-03 | 1.5E-02 | 1.5E-03 | 4.35E-05 | 8.02E-06 | 5.48E-05 | 5.48E-06 | 1.18E-02 | 2.19E-03 | 1.5E-02 | 1.5E-03 |  |
| SS9 | - | 1.73E-03 | 1.84E-02 | 1.73E-03 | - | 6.33E-06 | 6.75E-05 | 6.33E-06 | - | 1.73E-03 | 1.85E-02 | 1.73E-03 |  |
| Mean | 4.73E-03 | 5.31E-03 | 1.8E-02 | 1.76E-03 | 1.74E-05 | 1.95E-06 | 6.61E-05 | 6.47E-06 | 4.75E-03 | 5.33E-03 | 1.81E-02 | 1.77E-03 |  |
